# Artificial intelligence (AI)-powered diagnostic support for stroke via Telegram bot: preliminary findings

**DOI:** 10.1186/s12883-026-05065-3

**Published:** 2026-06-19

**Authors:** Babatunde Ogunmiloro

**Affiliations:** University College Hospital, Ibadan, Nigeria

**Keywords:** Artificial intelligence, Telegram bot, Stroke, Underserved regions, Deep learning, Transfer learning

## Abstract

**Background:**

Annually, stroke affects over 15 million people globally. Early intervention is critical in the management of stroke. However, the “golden hour” opportunity for timely intervention is often missed in underserved regions where experts in neuroimaging are scarce, with some countries having 1 per million inhabitants in comparison to high-income countries with 10 per million inhabitants. This study explores the feasibility of a lightweight AI-powered diagnostic support system for stroke deployed via a widely accessible platform (Telegram) to help clinicians in underserved settings.

**Methods:**

This study utilized a comparative analysis of two transfer learning models (EfficientNetB0 and MobileNetV2) which were employed for training, using 6,650 publicly available, anonymized but radiologist annotated brain CT images from three classes: normal (*n* = 4,427), hemorrhagic stroke (*n* = 1,093), and ischemic stroke (*n* = 1,130). An 80/20 split was executed at slice-level as a result of lack of patient identifiers which this study acknowledges as a limitation. A separately sourced Kaggle dataset with labels but no metadata on annotator provenance was used for quasi-external validation with further evaluation using standard performance metrics. The selected model was integrated into a Telegram bot (@BrainfloBot) with real-time inference capabilities, and automatic data deletion to ensure privacy compliance.

**Results:**

EfficientNetB0 demonstrated superior performance over MobileNetV2, achieving 99% accuracy (95% Cl: 98.2–99.5%) with excellent inter-rater reliability (κ = 0.985) during training and internal validation with precision and recall values exceeding 96% across all classes. While quasi-external validation using the balanced set, the selected model achieved a robust performance with 95% accuracy level (95% CI: 90.3%-97.9%, *n* = 150) with excellent agreement (κ = 0.925). For the unbalanced set, 97% accuracy level (95% Cl: 95.77%-98.03%) with excellent agreement(κ = 0.948) The model showed particularly strong performance in hemorrhagic stroke detection (98% precision, 96% recall), critical for preventing inappropriate thrombolytic therapy.

**Conclusions:**

These preliminary findings demonstrates a successful integration of a novel AI-powered stroke diagnostics with a widely accessible messaging platform. This system can potentially close neuroimaging gaps in underserved regions where neuroimaging specialists are scarce. However, the study limitations include lack of patient-level separation, annotation provenance for external validation dataset, possible selection bias and lack of real world testing. Future work can integrate real world validation, out of distribution handling and robust testing with clinical images.

**Graphical Abstract:**

**Figure:**
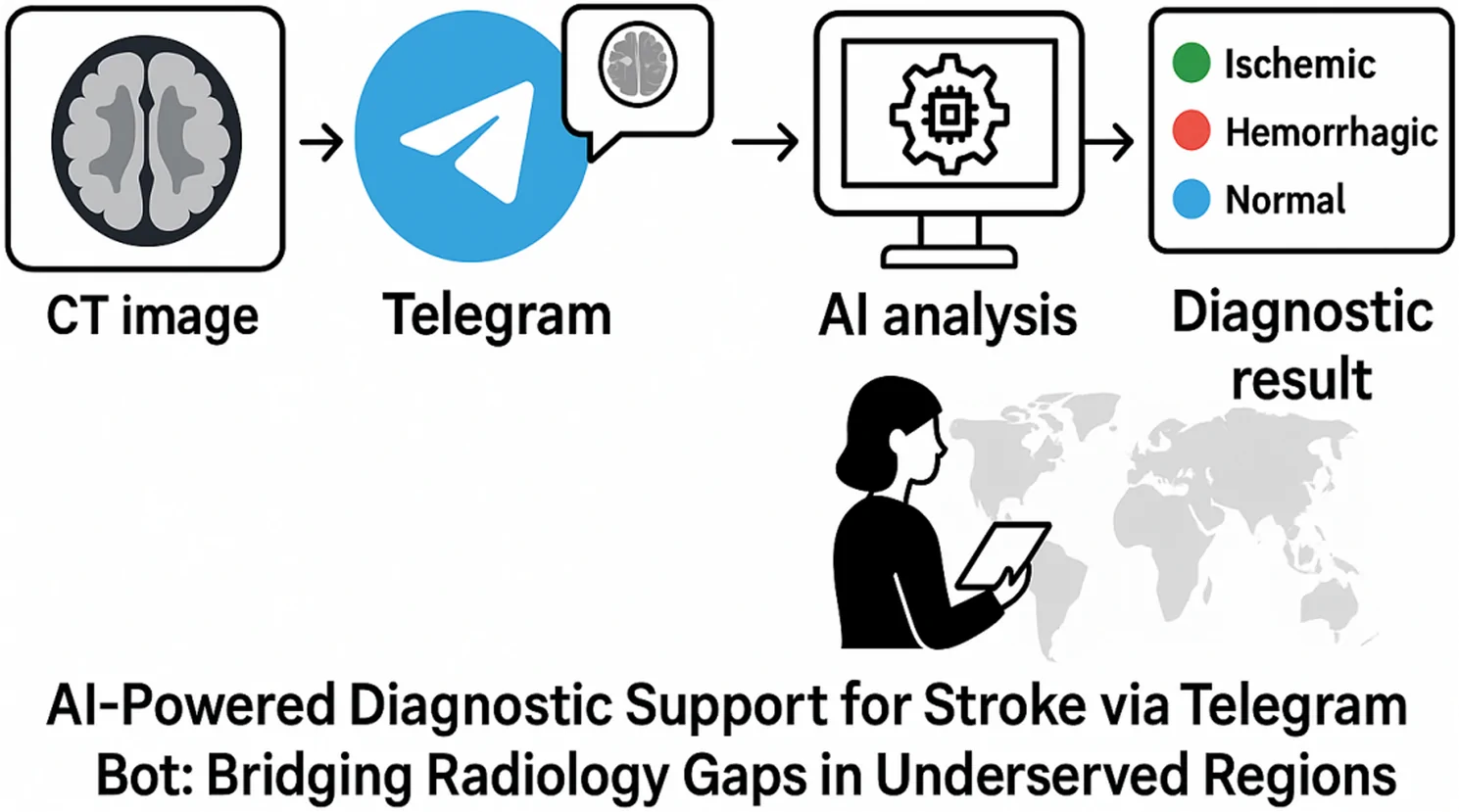
Graphical abstract of diagnostic workflow

## Introduction

According to the World Health Organization (WHO), 15 million people suffer a stroke annually, out of which 5 million die and another 5 million are permanently disabled significantly impairing quality of life and negatively impacting economic outcomes. Stroke also affects 8% of children with sickle cell disease [1]. According to the World Stroke Organization, there are over 12.2 million new strokes each year. Globally, one in four people over age 25 will have a stroke in their lifetime. Each year, over 16% of all strokes occur in people 15–49 years, over 62% in people aged 70 and above, 47% in men and 53% in women [2]. 

The WHO defines stroke as a focal (or at times global) neurological impairment of sudden onset, lasting more than 24 h (or leading to death), and of presumed vascular origin [3]. It remains the second leading cause of death globally. The concept of the golden hour rule applies to stroke management as the first 60 min after onset of symptoms represents the window period for the administration of therapy which can ultimately improve patient outcomes [4]. 

The global shortage of specialists in medical imaging especially in underserved regions, with some countries having alarming statistics of 1 neuroradiologist per million inhabitants vis-a-vis circa 10 per million inhabitants in high-income countries with better medical facilities and access [4]. This paucity extends to neuroimaging interpretation as brain computed tomography (CT) scans are often not interpreted in these regions [4]. This affects the capacity to distinguish between ischemic and hemorrhagic stroke cases which may increase mortality rates. This brings about the need to harness the power of AI in disease intervention.

Artificial Intelligence has shown remarkable progress in neuroimaging as seen in studies showing how machine learning and deep learning frameworks have been used to build predictive models that achieved accuracy levels comparable to diagnostic accuracies of radiological experts in detecting ischemic stroke, hemorrhagic stroke, and transformations [5, 6].

Integration of AI into neuroimaging improves early detection and promotes timely intervention [7, 8]. The reality of AI and its potential in diagnostics presents the chance to democratize access to expert-level diagnosis of stroke cases. AI tools have been utilized in the diagnosis of stroke in health care centers in high-income countries [9, 10], but use of these tools for detection in underserved regions comes with various challenges that range from lack of health care infrastructure, high cost, lack of access, and complexity in integration with existing systems [11, 12]. 

Telegram boasts over 950 million active users globally [13], and the utilization of its bot feature is well documented in literature [14, 15]. Its utility on smartphones, and easy to use features, make it suitable for use in underserved regions where stroke care is sub optimal [16]. The deployment of an AI model via telegram bot may promote access to a stroke diagnostic support system by millions of people worldwide. This democratization can provide a cost-effective approach to stroke management and access to stroke prediction by clinicians in underserved regions.

This study presents the training, evaluation, and integration of an AI-powered stroke diagnostic support system via a Telegram bot engineered to provide interpretations to brain CT images of suspected stroke patients. Two transfer learning models were employed for this study for comparative analysis, culminating in the adoption of the best-performing model. This study also describes the steps in model training, evaluation, quasi-external validation and how the model is engineered for real time use and prompt interpretation of results.

By merging this model with a Telegram bot, this innovative study showcases a novel approach with the potential to narrow diagnostic gaps in underserved areas, improving diagnostic speed, accuracy, equity in healthcare delivery, digital health, and patient outcomes [17]. 

## Materials and methods

### Overview

This feasibility study developed a lightweight AI-powered cerebrovascular disease diagnostic support system made accessible via a Telegram interface designed to support clinicians in underserved regions. An auto-crop feature was added and the Telegram bot was engineered to accept a cropped CT image of the brain in png or Jpeg formats, processed in seconds with outputs of normal, hemorrhagic and ischemic stroke. The backend of the bot is the stroke prediction model designed from the EfficientNetB0 framework, a pre trained deep learning model whose performance was superior to that of MobileNetV2 trained on the same dataset.

### Dataset structure and preprocessing

Dataset for model training and validation was sourced from Kaggle [18] and contains 6,650 axial CT images of the brain structured in 3 classes labeled 0,1,2. Classes 0, 1, 2 contain 4,427, 1,093, 1,130 normal, hemorrhagic, and ischemic and CT scan images of the brain respectively. This dataset was prepared for the AI in healthcare competition held in 2021 in Istanbul also curated and annotated by seven radiologists [19]. However, patient-level meta data were not provided and inter-annotator reliability metrics (e.g., κ-statistics) were unavailable preventing separation of 2D slices belonging to the same patient. Hence, an 80/20 split was performed at the slice level which may may cause data leakage.

A tailored approach was executed to ensure model robustness. From upload handling, image preparation, normalization, augmentation, and label encoding.

The preprocessing pipeline includes:

1. Auto-cropping feature: Using dynamic thresholding to automatically identify and extract the brain regions from CT scans. An adaptive threshold calculation (threshold = min_value + (max_value - min_value) x 0.15, with the inclusion of a bounding box detection with 10% padding to preserve anatomical features. However, no segmentation ground truth was available to validate this feature which may result in varying performance across images with low contrast.
2. Image Standardization: All brain CT images were resized to 224 × 224 pixels and converted to RGB format to ensure compatibility with the adopted pre-trained transfer learning model.
3. Data Augmentation: This study implemented data augmentation techniques like random horizontal and vertical flipping, minimal contrast and brightness adjustment, 90-degree rotations. The goal was to ensure model robustness and reduce the possibility of overfitting. A deterministic seed set to 42 to ensure reproducibility.

### Model architecture

For comparative analysis, two state of the art deep learning pre-trained models were employed:

1. MobileNetV2: A lightweight mobile friendly pre-trained model, known for its efficiency and high accuracy in image classification tasks [20].
2. EfficientNetB0: known for its wide applications in computationally constrained environments and highly rated for its high accuracy value and compound scaling efficiency [21].

Both pre-trained models were initialized with pre-trained weights and fine-tuned using:

1. Global average pooling layer
2. Dropout layer (rate = 0.4) for regularization
3. Dense classification layer with softmax activation for three-class prediction.
4. Adam optimizer with an initial learning rate of 1×10⁻⁴

To address class imbalance as seen in the majority distribution in the normal class and under representation of the hemorrhagic and ischemic classes, the following strategies were executed [22]:

- 1) Using sklearn’s compute_class_weight function formula: w_i = N / (K * n_i)
  - N = total number of samples
  - K = number of classes
  - n_i = number of samples in class i
- Calculated class weights: {Normal: 0.50, Hemorrhagic: 2.03, Ischemic: 1.96}
- 2) Split ratio of 80:20 for train and validation to maintain class distribution.Training set: 5,320 images and validation set: 1,330 images.

**Fig. 1:**
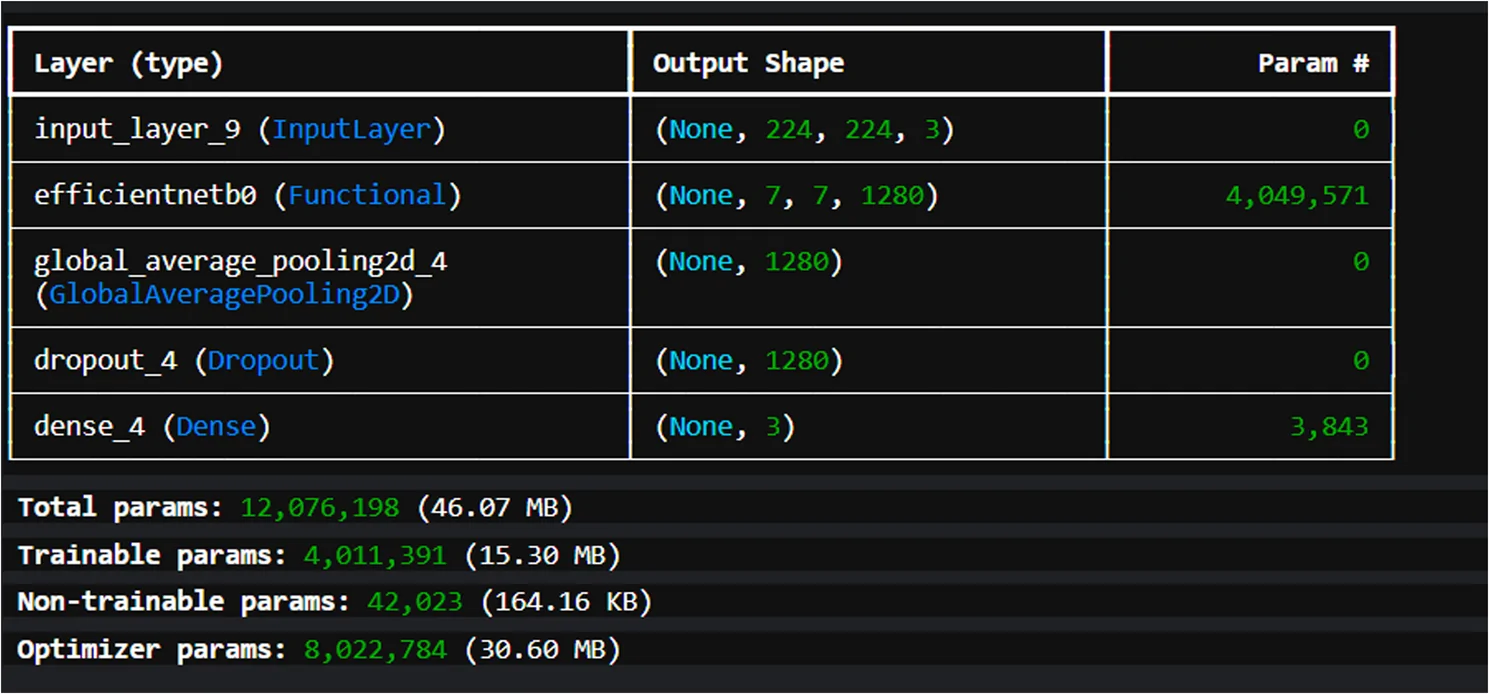
Summary of the EfficientNetB0 model layers with global average pooling and dropout to prevent overfitting. The final dense layer optimized for a multi-class prediction

The model architecture is seen in Fig. 1 below.

### Model training

Both models were trained for up to 50 epochs with early stopping (patience = 10) implemented if validation accuracy does not improve. Callback features implemented include:

1. Model checkpointing to save best-performing weights
2. Learning rate reduction on plateau (factor = 0.2, patience = 5)
3. Minimum learning rate threshold of 1×10⁻⁷

This study utilized Tensorflow and Keras libraries with Kaggle GPU acceleration enabled. Integrated development environment (IDE) was Kaggle notebook.

### Evaluation metrics

The models were evaluated using the following parameters:

Accuracy, precision, recall, f1-score (per class and macro-averaged), confusion matrix, visual plots.

### Telegram bot architecture

The bot was created at the @botfather interface of Telegram with the username @BrainfloBot. The ease of accessibility of Telegram in under served regions informed the decision to utilize it in democratizing AI diagnostic support systems in these regions. The architecture includes:

1. Telegram bot API (python-telegram-bot v13.7) which provides accessibility across mobile devices for user interaction and brain CT image upload serving as front-end.
2. Backend processing by the adopted EfficientNetB0 model.
3. Image processing: Accepts brain CT images in png or jpeg formats and with the auto cropping feature, RGB conversion, resizing uploads to 224 × 224 and a focus on 20% minimum brain area.
4. Prompt response generation feature after an upload. Response includes a prediction report with confidence intervals, class-specific probability distributions for all categories.
5. Security and privacy concerns: Telegram is not a Health Insurance Portability and Accountability Act (HIPAA) compliant platform or Food and Drugs Administration (FDA) approved; thus this diagnostic system is strictly a research proof-of-concept and not intended for clinical deployment.
6. Command Structure: The essential commands include /start: for support system initialization and display of instructions with welcome message.

### Quasi-external validation dataset and strategy

For the rigorous assessment of the adopted model, a quasi-external validation was conducted using a publicly available dataset also outsourced from Kaggle but with no annotation provenance, no information on radiologist involvement, no patient-level data and meta data on scanning procedure [23]. For maximum predictive value, a strict image filter was applied which resulted in the creation of a balanced and unbalanced datasets at slice level with n = 150 and 903, respectively. However, when sample size was increased to 500 per class, only 199 and 204 high-quality images from the hemorrhagic and ischemic classes passed the filter check. This dataset was not used during model training or internal validation to ensure true generalization and robustness of the model.

Batch inference and probabilistic softmax output were used for prediction set up to derive the class label and confidence level.

Evaluation Metrics: Standard evaluation metrics like accuracy, precision, f1-score, recall were incorporated.

For visualization: ROC curves and confusion matrices.

## Results

### Model training and evaluation metrics

The performance of the two models; MobileNetV2 and EfficientNetB0 were evaluated with key metrics which like precision, recall, F1-score and accuracy with EfficientNetB0 achieving a slightly higher accuracy level of 99% while MobileNetV2 achieved 98%. The synopsis are presented in Table 1 below.

**Table 1:** Evaluation metrics of MobileNetV2 and EfficientNetB0 for stroke classification

Model	Class	Precision	Recall	F1-Score	Support	Accuracy
MobileNetV2	**Normal**	**0.98**	**0.99**	**0.98**	**885**	
	**Hemorrhagic**	**0.99**	**0.95**	**0.97**	**219**	
	**Ischemic**	**0.95**	**0.96**	**0.96**	**226**	
	**----**	**-----**	**----**	**-----**	**1330**	**0.98**
	**Macro Avg**	**0.97**	**0.97**	**0.97**	**—**	
	**Weighted Avg**	**0.98**	**0.98**	**0.98**	**—**	
EfficientNetB0	**Normal**	**0.99**	**0.99**	**0.99**	**885**	
	**Hemorrhagic**	**0.99**	**0.97**	**0.98**	**219**	
	**Ischemic**	**0.97**	**0.96**	**0.97**	**226**	
	**-----**	**-----**	**—**		**1330**	**0.99**
	**Macro Avg**	**0.98**	**0.97**	**0.98**	**1330**	
	**Weighted Avg**	**0.98**	**0.98**	**0.98**	**1330**	

Narrative Summary of Key Findings are under Internal validation, Quasi-external validation and clinical interpretation.

### Internal validation

EfficientNetB0 mildly outperformed MobileNetV2 consistently across metrics:

1. 99% accuracy
2. Achieved precision/recall value > 96% across classes
3. Hemorrhagic class with the highest performance and Ischemic class with the lowest which may be due to subtle early changes

### Quasi-external validation

The EfficientNetB0 was quasi-externally validated and produced the following outomes:

For Balanced dataset (*n* = 150).

1. 95% Accuracy
2. Hemorrhagic class showed the strongest performance ( F1 = 0.97) while the ischemic class showed the lowest (F1 = 0.92) which may also be caused by subtle early changes seen at the onset of Ischemic stroke.

For Unbalanced dataset (*n* = 903).

1. 97% accuracy (may have been slightly inflated by class imbalance)

### Clinical interpretation

1. Hemorrhagic stroke detection is highly reliable
2. Ischemic class is the most challenging to predict
3. Dataset homogeneity could be a factor contributing to the high performance.

Caution should be applied in the interpretation of all evaluation metrics due to a potential data homogeneity across the split and both datasets for this study are from Kaggle repositories which may not fully represent clinical real world datasets.

The summary of the results of the external validation I carried out can be seen in Tables 2, 3 and 4 below.

**Table 2:** Evaluation metrics for external validation of the adopted EfficientNetB0 model (balanced set)

Class	Precision	Recall	F1-Score	Support (*n*)
Normal	**0.96**	**0.94**	**0.95**	**50**
Hemorrhagic	**0.98**	**0.96**	**0.97**	**50**
Ischemia	**0.90**	**0.94**	**0.92**	**50**
Accuracy	**—**	**—**	**0.95**	**150**
Macro Avg	**0.95**	**0.95**	**0.95**	**150**
Weighted Avg	**0.95**	**0.95**	**0.95**	**150**

**Table 3:** Evaluation metrics for external validation of the adopted EfficientNetB0 model (unbalanced dataset)

Class	Precision	Recall	F1-Score	Support
Normal	**0.99**	**0.96**	**0.98**	**500**
Hemorrhagic	**0.99**	**0.98**	**0.98**	**199**
Ischemia	**0.89**	**0.98**	**0.94**	**204**
Accuracy	**---**	**---**	**0.97**	**903**
Macro Avg	**0.96**	**0.97**	**0.98**	**903**
Weighted Avg	**0.97**	**0.99**	**0.98**	**903**

**Table 4:** Comparative performance metrics of the EfficientNetB0 model on balanced vs. unbalanced external validation sets

Metric	Unbalanced (*n* = 903)	Balanced (*n* = 150)	Interpretation
Accuracy	**97%**	**95%**	**Slightly higher in the unbalanced set. May reflect over-representation of the Normal class.**
Macro Avg F1-score	**96%**	**95%**	**Higher in unbalanced due to large support; balanced is slightly lower but fair across all classes.**
Normal Precision	**99%**	**96%**	**Inflated in unbalanced due to dominant class.**
F1-score Hemorrhagic	**98%**	**97%**	**Consistently strong.**
Ischemia F1-score	**94%**	**92%**	**Model performs comparably well on this challenging class.**

### Visualizations for training and evaluation

This section shows the visualization techniques of the models employed for this study during training, evaluation and external validation as shown in Figs. 2, 3, 4, 5, 6, 7, 8, 9, 10 and 11 below.

**Fig. 2:**
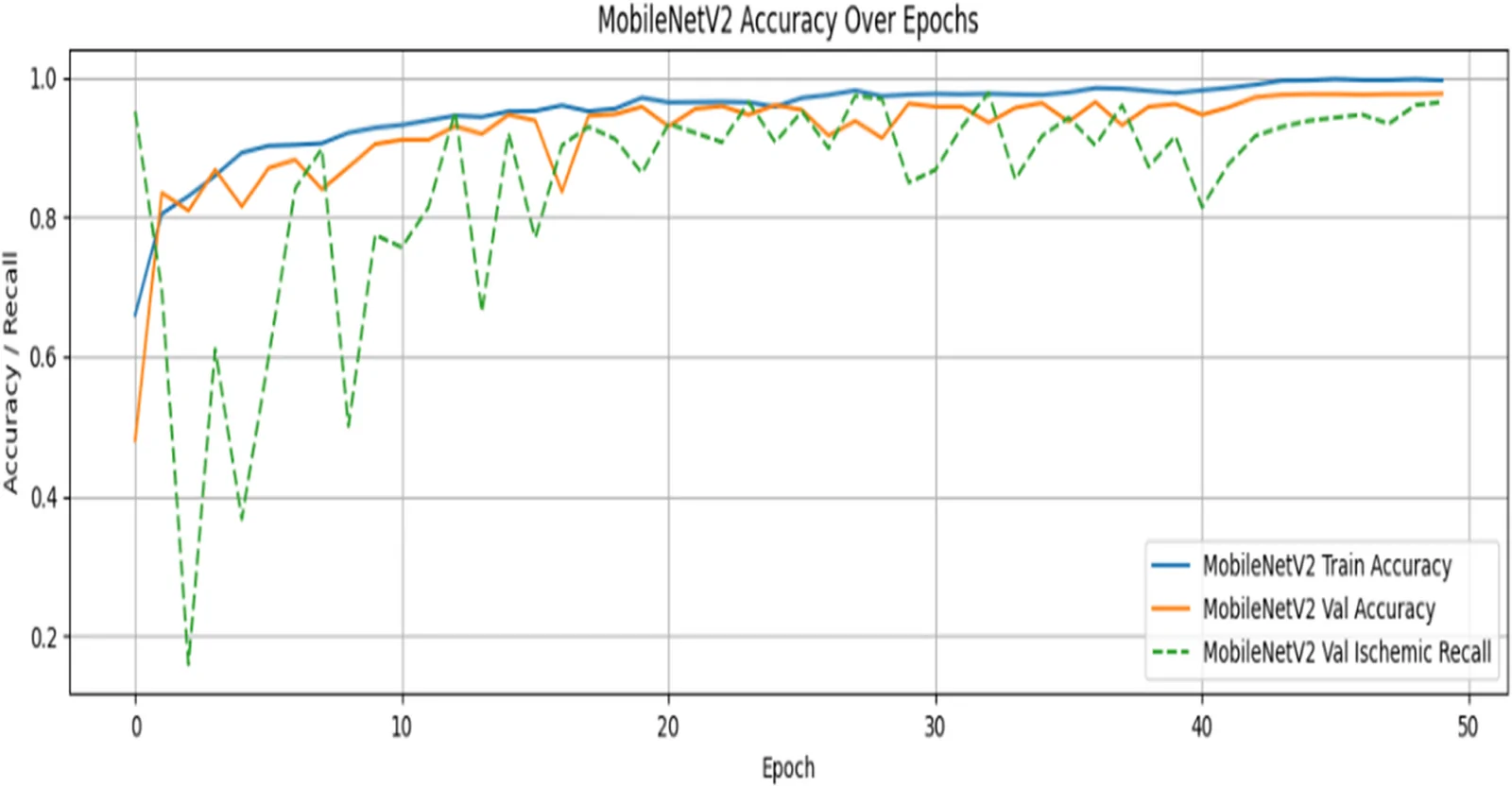
Training and validation accuracy curves for the MobileNetV2 showing validation accuracy closely following the training accuracy throughout the epochs with a steady increase in value which signifies the model is learning well

**Fig. 3:**
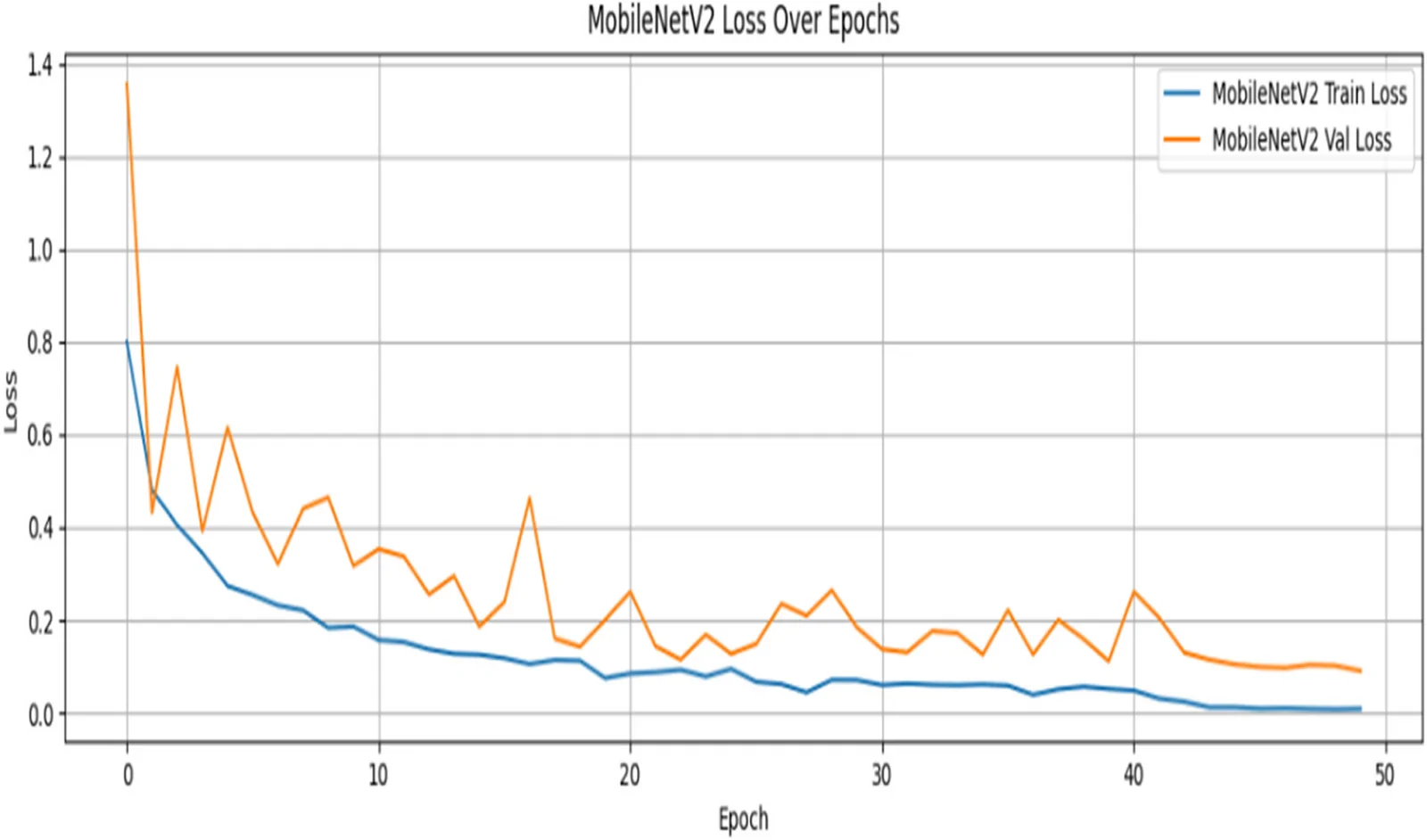
Training and validation loss for MobileNetV2. The loss curves show good generalization by the model, with validation loss closely following the training loss with a steady decline in loss values

**Fig. 4:**
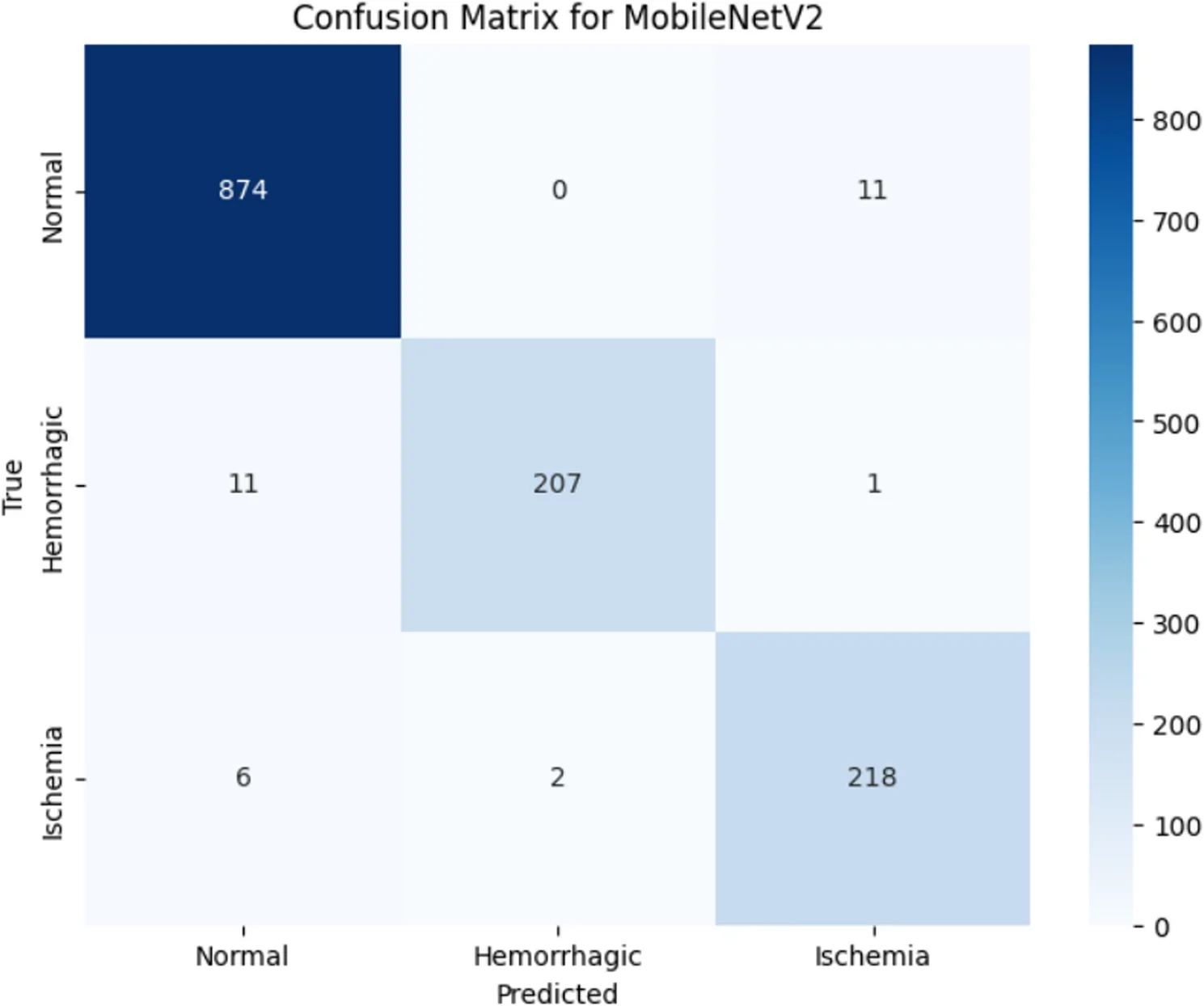
Confusion matrix for MobileNetV2 which reveals excellent classification performance with minimal misclassification across the three classes with the normal class emerging with a near perfect result followed by the hemorrhagic and ischemic classes. The model’s displayed reliability with very low false positives and negatives across the classes

**Fig. 5:**
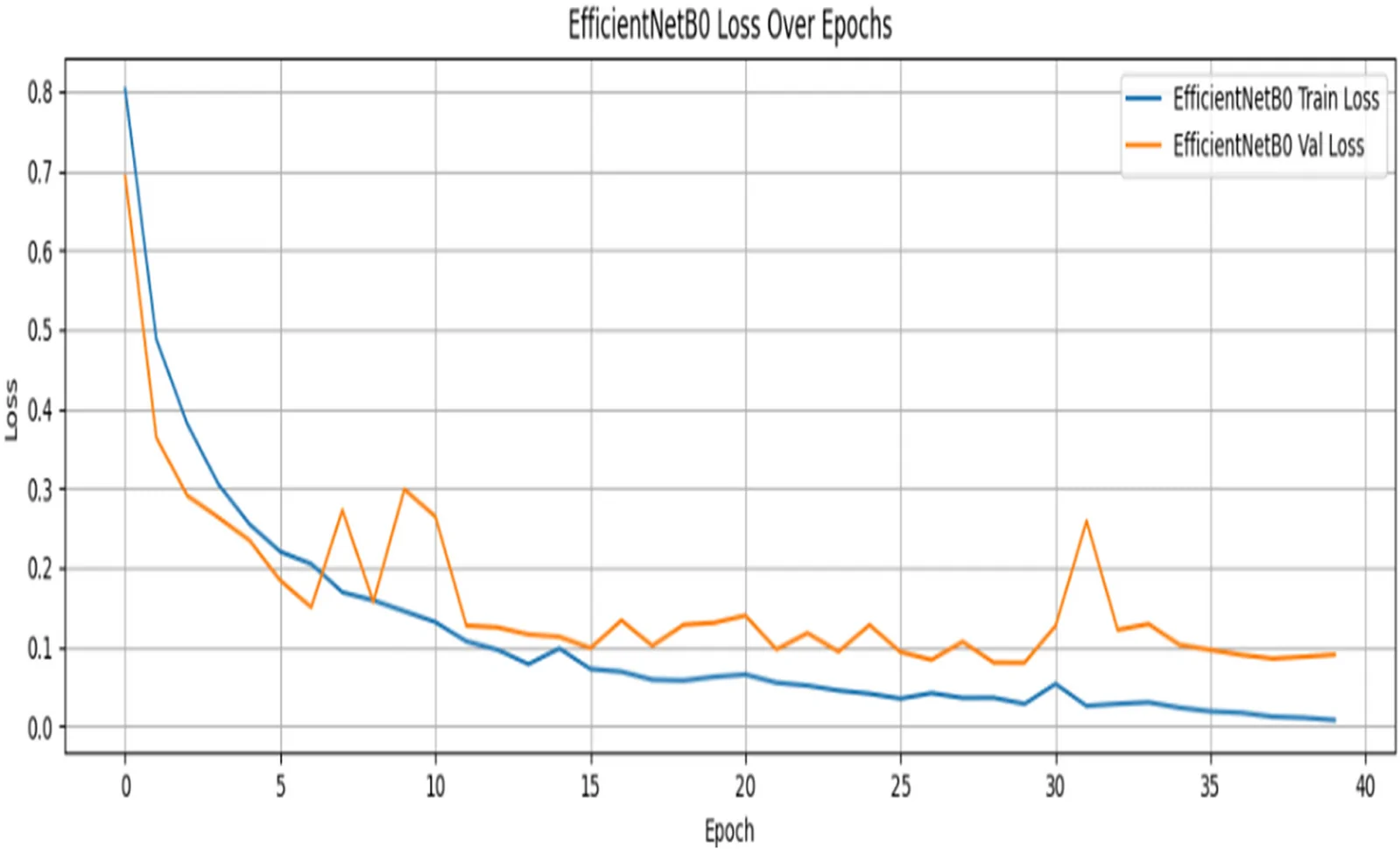
Training and validation loss curves for EfficientNetB0 showing loss over epochs with a steady decline in loss values and validation loss closely following training loss. There is an isolated spike in loss value for validation loss which may suggest a potential overfitting or misclassification but got normalized towards the end

**Fig. 6:**
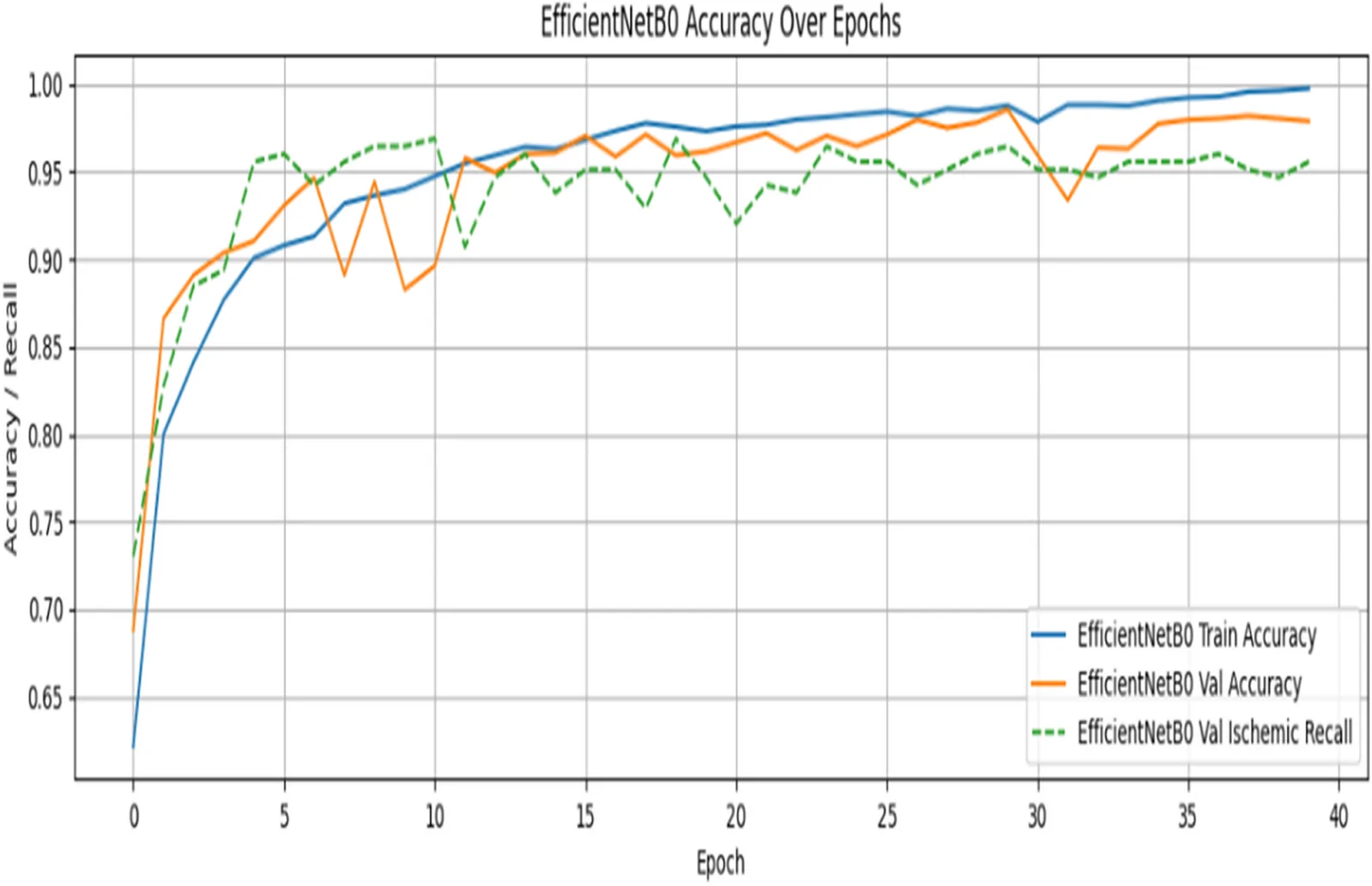
Training and validation accuracy curves over epochs for EfficientNetB0 showing the training and validation accuracy curves steadily increasing which signifies how well the model generalizes

**Fig. 7:**
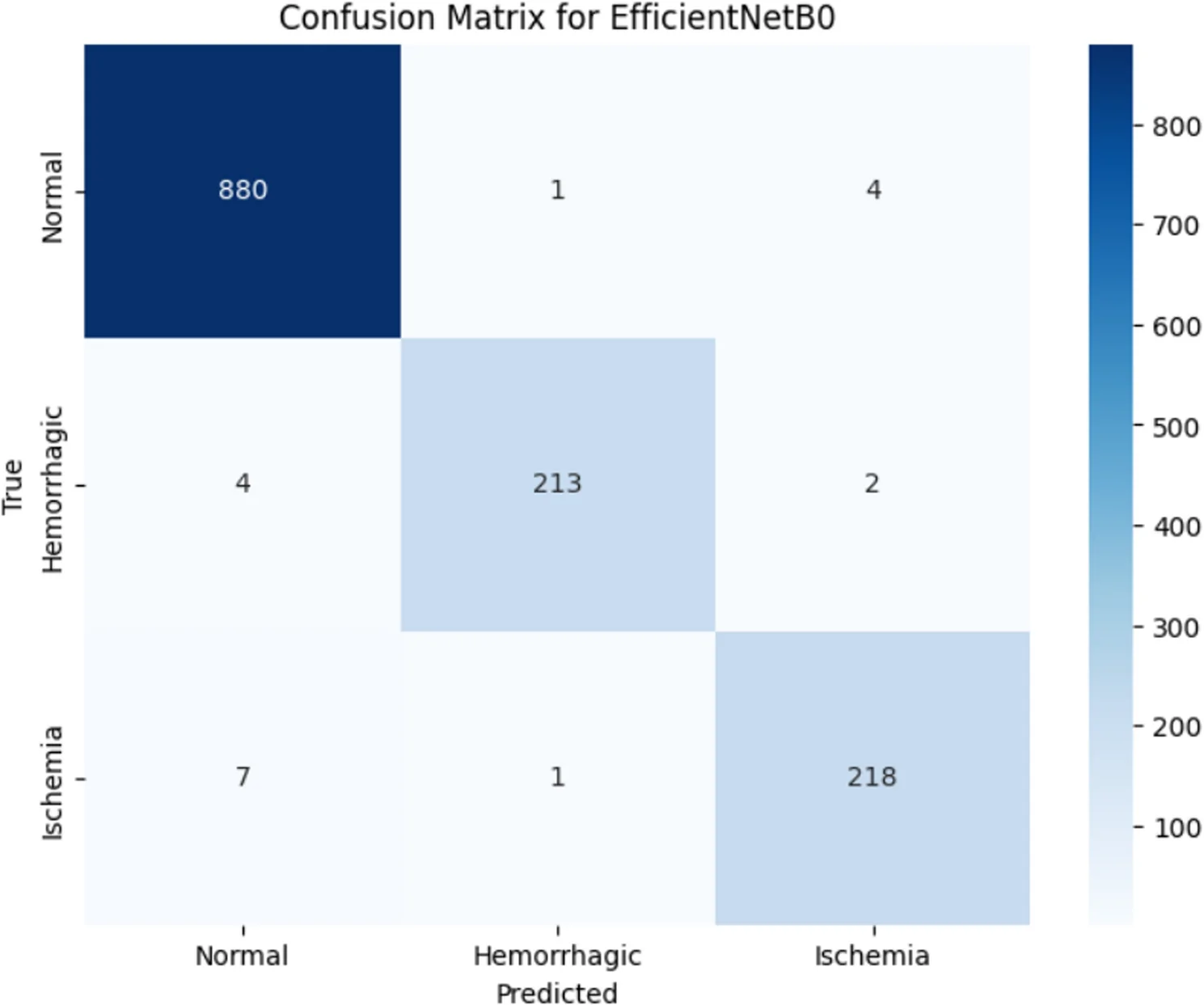
Confusion matrix for EfficientNetB0 demonstrates a superior classification accuracy across classes compared to the MobileNetV2, the model recorded a higher number of correct classifications across the three classes which justifies the adoption of the model for integration with the telegram bot

### Visualizations for external validation

#### For unbalanced set (Figs. 8 and 9)

**Fig. 8:**
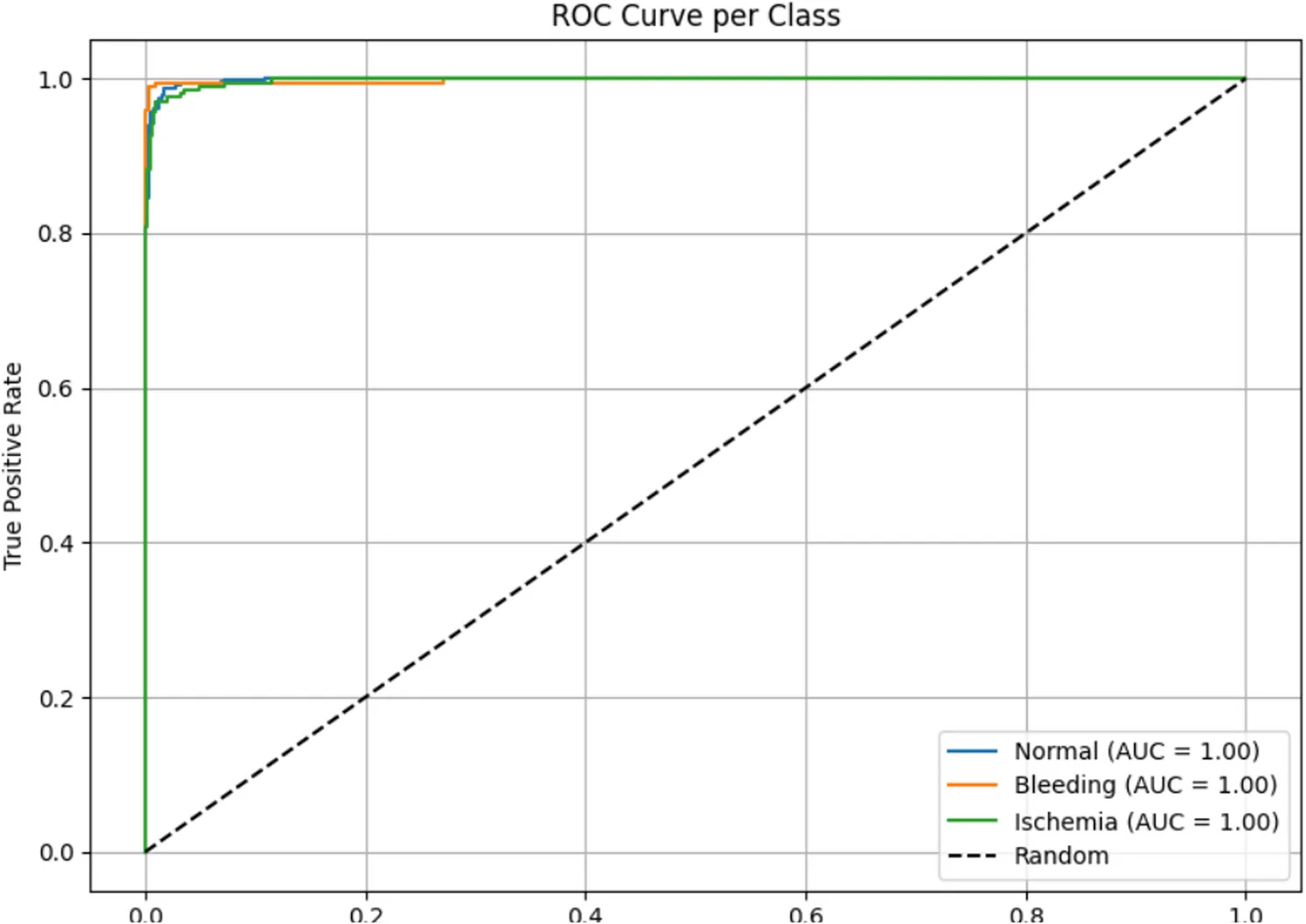
ROC curves for external validation using the unbalanced dataset. The model demonstrates strong discriminative performance, with AUC values above 0.95 across all classes. The hemorrhagic class shows the highest AUC, followed by the normal and ischemic classes, despite class imbalance. These findings indicate good performance on an independent, curated dataset; however, they do not constitute real-world clinical validation, and clinical applicability must be confirmed in a prospective clinical setting settings

**Fig. 9:**
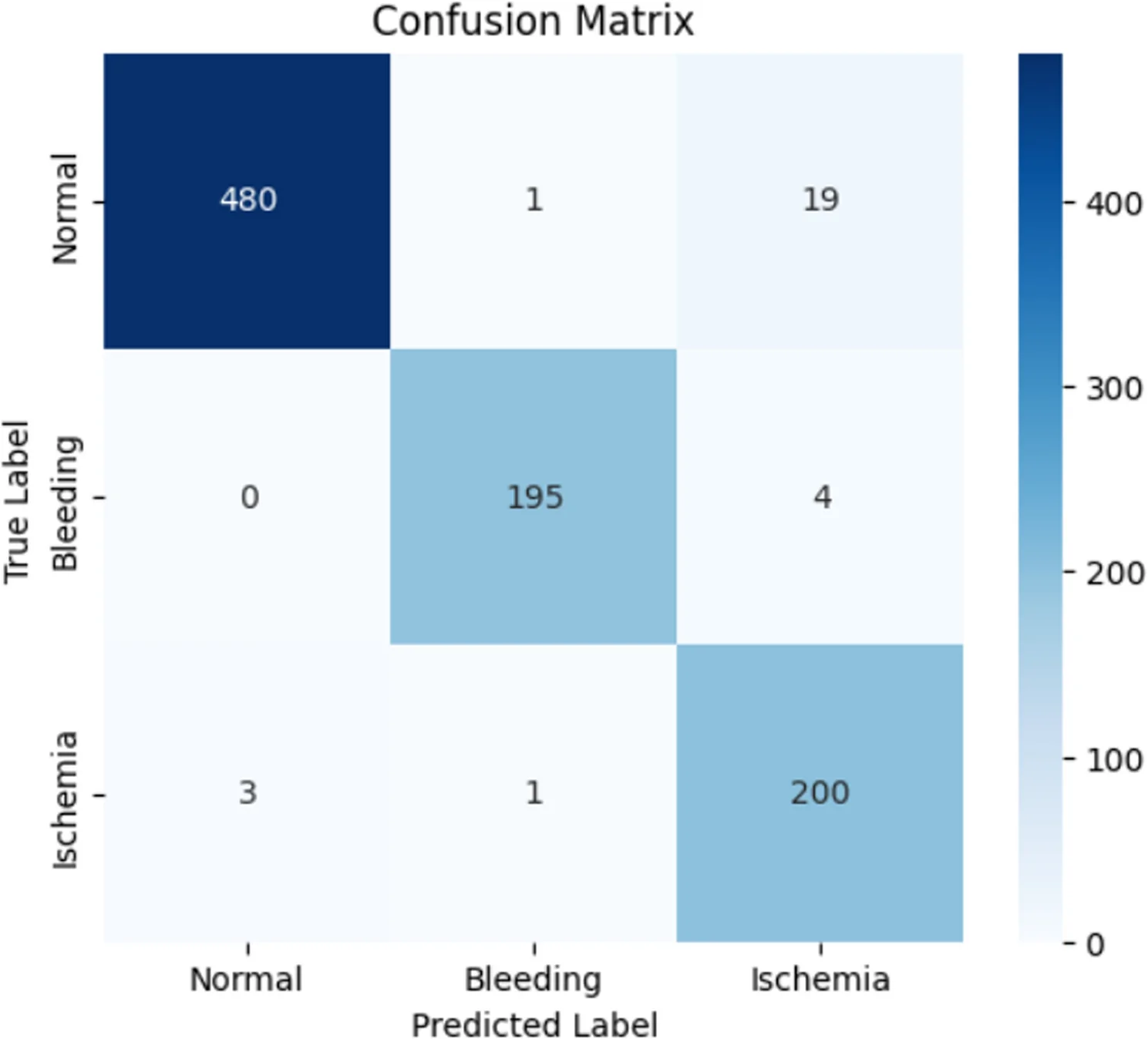
Confusion matrix for external validation (unbalanced set) demonstrate low misclassifications across classes with the ischemic class being the least. This reflects the class imbalance in the external validation set. The true classifications demonstrate a strong predictive value for unseen data

#### For balanced set (Figs. 10 and 11)

**Fig. 10:**
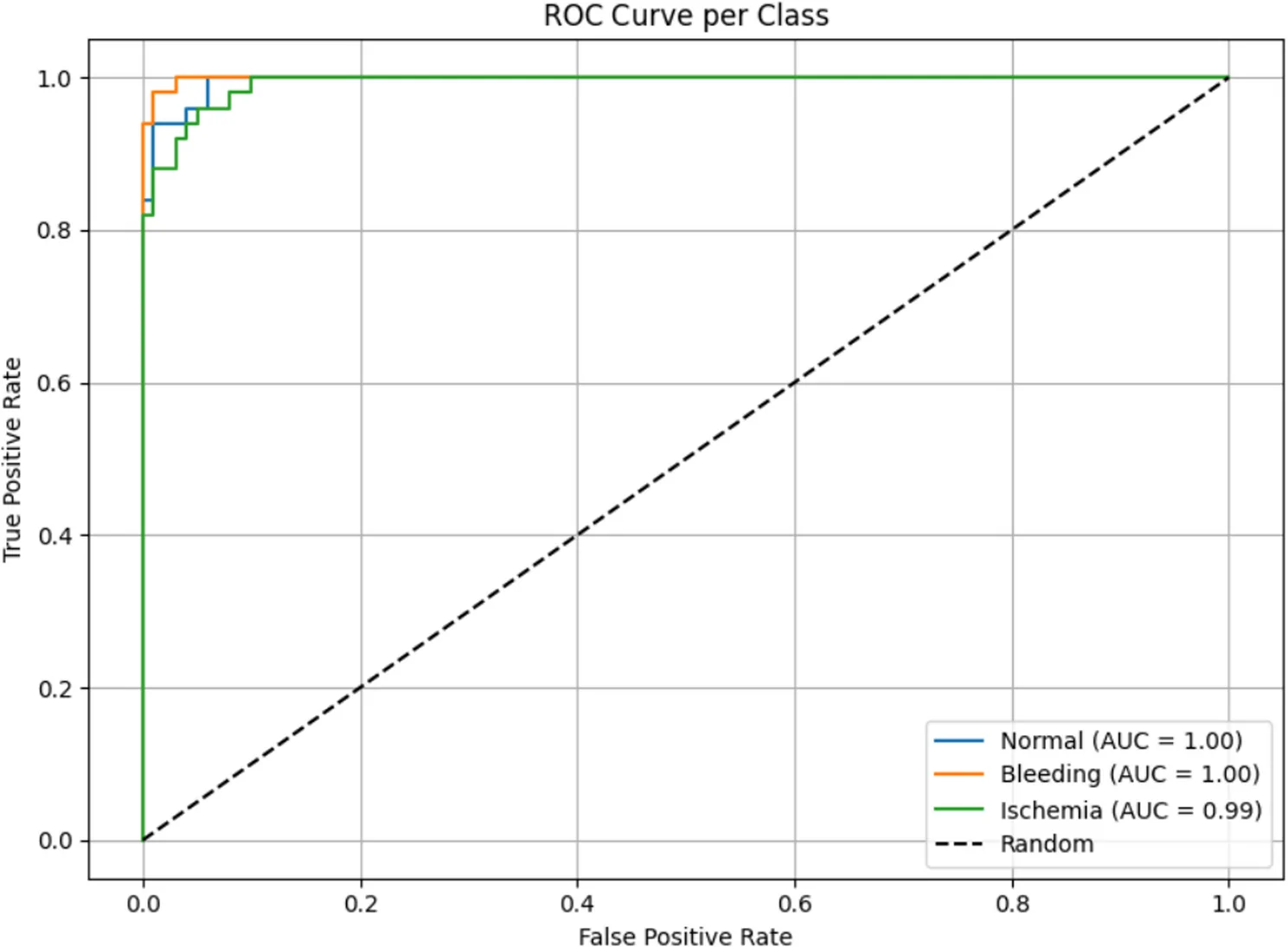
ROC curves for external validation using the balanced dataset. The model demonstrates strong discriminative performance across all classes, with AUC values exceeding 0.90. The hemorrhagic class shows the highest AUC, followed by the normal and ischemic classes. These results indicate good performance on an independent, curated dataset; however, they should not be interpreted as evidence of clinical utility, as real-world prospective validation is still required

**Fig. 11:**
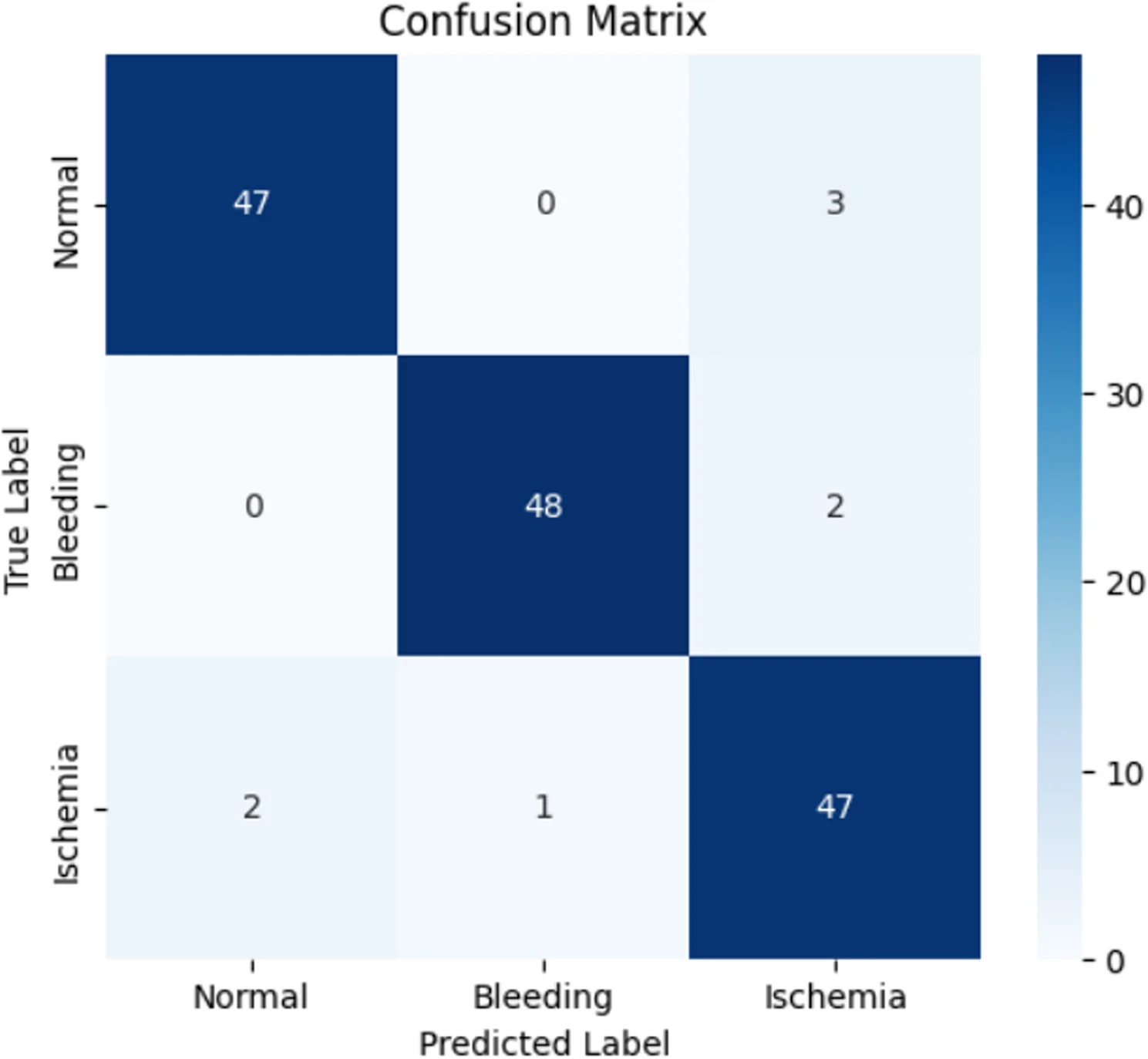
Confusion matrix for the external validation (balanced set). The model shows strong per-class discrimination across normal, hemorrhagic, and ischemic categories on an independent, curated dataset. These results highlight consistent performance under balanced conditions; however, they do not indicate suitability for clinical decision support, as real-world, prospective clinical validation is still required

## Discussion

The comparative analysis between the two models shows a significant difference in their overall performance with a slight superiority displayed by EfficientNetB0 across all classes. The implications of these nuances include:

1. Diagnostic Accuracy in Underserved Regions: The overall slight superiority of EfficientNetB0 across all classes indicates its capacity for a robust feature representation of the dataset used for this study. Its emergence as the best-performing model, with very high accuracy levels shows how promising it is as a diagnostic support system pending clinical validation. This tool can also assist in the prompt identification of patients eligible for thrombolytic therapy upon validation in real world.
2. Clinical Implication Of Performance Metrics: Both models achieved exceptionally high precision values across all classes. This indicates a very low false positive rate which is significant in the diagnosis of stroke cases and can facilitate early intervention.

Very high recall values recorded by both models signifies strong sensitivity to stroke cases and also strong capacity to differentiate stroke cases from non stroke cases. However, EfficientNetB0 demonstrated stronger recall value suggesting a slight superiority over MobileNetV2. A 0.97 recall value for EfficientNetB0 shows a higher tendency to identify intracranial hemorrhage on CT scan images of the brain which is the the most radiologically distinct type of stroke. A higher value of 0.96 recall value for ischemic cases is significant for diagnosing ischemic cases. It’s noteworthy that sometimes, early ischemic changes are often subtle and harder to discern by human experts.

3) Diagnostic performance on External dataset: The overall validation accuracy level of 95% achieved on the external dataset, with very high f1-scores of 0.95, 0.97, and 0.92 for Normal, Hemorrhagic and Ischemic classes respectively suggests potential usefulness for diagnostic support and for the purpose of triage for clinicians in underserved regions. The strong metrics of performance achieved by the model demonstrate its excellent discriminative ability for stroke cases.
4) Scope of Use: This tool was intended to serve as diagnostic assistants in hospitals in remote areas where specialists are lacking but with a CT scan or areas where CT scans are present but the specialists present are overwhelmed with making interpretations and meeting a deadline. The goal of invention of this tool is tailored towards a complementary role rather than an outright replacement of specialists.

This tool can also serve as educational and training systems to educate primary health care workers on stroke diagnosis and management.

5) Clinical Safety Risks include:
  i) The most critical risk lies in the misclassification of hemorrhagic as ischemic stroke which may lead to inappropriate thrombolysis which can worsen stroke outcomes.This risk should be examined by future works.
  ii) Early ischemic changes may also be subtle and remain undetected.
  iii) Telegram image uploads vary widely
  iv) Medico-legal issues can arise from Telegram HIPAA non compliance
6) Out of Distribution Behavior: Brain CT scans reveal other pathologies like brain tumors, calcifications, infections etc which were not covered by the training data constitute out-of distribution inputs for the model. This consequently means the model will return an output for OOD uploads. This underscores the need for a future “Others” category prior to clinical deployment.
7) Picture Archiving And Communication Systems (PACS): In most low and middle income countries, many hospitals have partial PACS or no PACS at all making most clinicians to frequently rely on exported images, screenshots, flash drives for sharing images. This makes this study germane and aimed at underserved regions.Hence, clinicians can upload Jpegs or png formats of CT images on the Telegram bot for prompt interpretation pending model integration with the PACS of any health facility in the future.
8) Domain Shift: For the clarity purpose, the Telegram bot was tested with mobile captured CT images of different stroke classes which may be considered noisy images on a small scale and it successfully predicted an output correctly in most cases.

### Related works

In my examination of other related works, I found a study that trained a Convolutional Neural Network model on structured dataset containing clinical information of stroke patients and achieved an accuracy level of 95%, which clearly shows the underutilization of CNNs as they perform better on datasets containing radiological images like x-rays and CT scans. The study compared their CNN model performance with Machine Learning frameworks like Logistic Regression, Naive Bayes, Random Forest and Support Vector Machine which all achieved similar accuracy levels between 87 and 95% [24]. In comparison with this study which achieved higher accuracy level of 98% and this study successfully integrated the model developed with a Telegram bot to democratize stroke diagnostic support.

Another study utilized the EfficientNetB0 as their adopted model in comparative analysis with other transfer learning models like MobileNetV2, ResNet101 and GoogleNet and achieved a very high accuracy level of 97.93% but failed to test the application of their model [25].

A particular study also developed a chatbot that utilizes only demographic data as user input in stroke prediction using deep learning framework. This study failed to state in their approach how their reliance on demographics alone guarantees model robustness and generalizability [26].

The majority of literature on model development in the health space never gets beyond the model development stage with varying accuracies, most fail to test the real world applicability of their models as exemplified in these related works [27–29].

This model can potentially bridge diagnostic divides and address the radiological gaps in underserved regions by serving as an important clinical triage assistant for stroke cases.

### Limitations

1. Incomplete 24-hour Deployment of The Telegram Bot: The bot, although fully integrated with the predictive model is currently hosted by the Kaggle IDE which does not guarantee a 24 h runtime. A cloud based deployment will be incorporated in the future to ensure a 24 h accessibility to the predictive features.
2. Potential Limited Dataset Diversity: The dataset used for this innovative study was drawn from public repositories like Kaggle which did not include metadata regarding the number of annotators, labeling protocol, or inter-annotator agreement. The absence of this information limits the ability to verify annotation reliability and introduces uncertainty regarding label noiser [30].
3. Absence of Integrated Explainability in the Bot Interface: While the model demonstrates excellent predictive performance, it does not currently execute a Grad-CAM feature which can promote transparency and explainability. This may limit trust amongst clinicians [29].
4. Sample Exclusion due to Preprocessing criteria: During preprocessing, an image filter was applied on the external dataset and also on the model to ensure maximum predictive result via a better exposure of the model to a minimum brain area of 20%. This automatically exclude CT images with brain area less than 20%. This may create a bias towards images with clearer and more exposed brain area [31].
5. Data Quality and Integrity: The quasi external validation dataset used for this study lack input of experts in annotations and labeling of brain CT images which can potentially inflate the accuracy level recorded for quasi-external validation.
6. Class Imbalance in External Validation set: The emergence of this imbalance reflects the relative paucity of high quality annotated brain CT images especially in the hemorrhagic and ischemic classes. This outlines a real world scenario where normal brain CT scans can outnumber the pathologic ones in clinical repositories. This can pose a real challenge in model development.
7. Further optimization: As with other AI predictive models that are designed to return a predictive output, the model developed by this study will require further technical tweak to prevent it from predicting non stroke CT images as it may cause confusion for these cases. Future studies can work on this aspect.
8. Human Experts Comparison: This study acknowledges the lack of benchmark comparison with human experts like radiologists or neurologists as the absence of these experts can limit the utility of this model.
9. Auto crop feature: The auto-crop feature has not been clinically validated and may introduce cropping errors that affect classification tasks.
10. Slice-level Redundancy and CT Variability: The use of CT slices rather than patient-level split, adjacent slices may share similar visual trends making the risks of slice-level redundancy to exist and and generalizability inflation. The dataset used represents curated CT slices with limited variability in quality, artifacts, and other parameters which may as well limit the model’s capacity for noisy data.
11. Medico-legal Considerations of Telegram HIPAA Non Compliance: Telegram is not HIPAA-compliant, and the implications of an exchange of clinical images can attract legal tussles. Hence, this tool should be used as a support system and not a replacement for formal diagnostic procedures.
12. Domain Shift and Validation: Although the Telegram bot successfully processed CT images uploaded via real-world pathways (screenshots, exported JPEG/PNG images, and smartphone photographs), this study acknowledges that these functional tests do not constitute a formal evaluation of model performance under domain shift. The model has not yet been robustly validated on noisy, low-resolution, or variably windowed mobile-acquired images. This represents an important limitation, and future work will include structured robustness testing on prospectively collected real-world clinical images.

### Future works

1. Further validation using expert annotated datasets to reinforce model robustness.
2. Prospective Clinical trials with real patients.
3. Transition from 2D slice based analysis to 3D so as to eliminate slice-level redundancy.
4. Integration of Explainability features like Grad-CAM, saliency maps to promote trust and transparency amongst clinicians.
5. Future studies should consider integration of cross validation for more statistical rigor.
6. Auto-crop validation will be invaluable for future studies to ensure consistent performance across low-contrast and nosiy CT images.
7. Out of distribution integration for classification tasks should be considered to prevent diagnostic dilemmas.
8. Future studies should consider Grad-cam saliency features to promote explainability.
9. In the short term, clinicians can upload JPEG/PNG exports or smartphone photos of CT scans. As future work, the model could be integrated directly into hospital PACS infrastructure via DICOM or DICOMweb interfaces to enable seamless clinical workflow integration.
10. Future studies can explore the diagnosis and the relationship between white matter alterations rate and cognitive impairment after an acute stroke [32].

This study exemplifies the possibility of utilizing an AI powered diagnostic support system to classify stroke CT images into the aforementioned three classes. The high accuracy results achieved in this feasibility study points to the capacity of this model to serve as diagnostic support for clinicians in underserved regions, serve as an educational and training tool, potentially narrowing neuroradiology gaps.

## Conclusion

This study exemplifies the possibility of utilizing an AI powered diagnostic support system to classify stroke CT images into the aforementioned three classes. The high accuracy results achieved in this feasibility study points to the capacity of this model to serve as diagnostic support for clinicians in underserved regions, serve as an educational and training tool, potentially narrowing neuroradiology gaps.

### Ethical considerations

No breach of confidentiality occurred, with all data fully anonymized in accordance with ethical AI research standards. The open-source Kaggle dataset ensured compliance with data privacy regulations. To mitigate AI bias, stratified data augmentation and class-weighted loss functions was applied to balance representation across all classes. Additionally, the Telegram bot was designed with end-to-end encryption to secure user-uploaded brain CT images and diagnostic outputs, adhering to data protection guidelines. Continuous monitoring for bias and security vulnerabilities will be prioritized in future validations to maintain trust and equity in deployment, particularly in underserved regions.

## Data Availability

The dataset used for model training and internal validation—comprised of 6,650 CT images spanning normal, hemorrhagic, and ischemic classes—was obtained from the Kaggle repository by ozguraslank at https://www.kaggle.com/datasets/ozguraslank/brain-stroke-ct-dataset. The independent dataset used for external validation (balanced and unbalanced brain CT sets) was also obtained from Kaggle, via https://www.kaggle.com/datasets/mouhsinenaghach/brainstrocksplit.
